# Managing intra-operative complications during totally extraperitoneal repair of inguinal hernia

**DOI:** 10.4103/0972-9941.27732

**Published:** 2006-09

**Authors:** Davide Lomanto, Avinash N Katara

**Affiliations:** Department of Surgery, Minimally Invasive Surgical Centre, Yong Loo Lin School of Medicine, National University of Singapore, Singapore

**Keywords:** Complications, inguinal hernia, inguinal hernia repair, laparoscopic surgery, total extraperitoneal

## Abstract

Laparoscopic inguinal hernia repairs are looked upon as technically demanding procedures having have a stiff ‘learning curve’ associated with its performance in terms of clinical outcome and patient's satisfaction. Complication rates have been shown to drop with increased surgical experience. The complication rate for laparoscopic repair of inguinal hernia ranges from less than 3% to as high as 20%. Complications of a totally extraperitoneal (TEP) repair include general complications that occur with any surgical procedure and anesthesia, mesh-related complications and those specific to the TEP procedure, like visceral injury, vascular injury, nerve injury and injury to the cord. Intraoperative complications can occur at every step of the operation, even though some of them are only occasionally reported. However, it is important to analyze all of them chronologically, so that we can define methods to prevent them or tackle them if they occur. Risk reduction strategies are required to improve the clinical outcome of TEP and this must be adopted for each individual surgical step.

Total extraperitoneal (TEP) repair for inguinal hernia was first reported in 1993.[1] It has evolved to become a popular method of endoscopic repair of inguinal hernias. It involves placement of a prosthetic mesh in the preperitoneal space to cover all potential hernial sites in the myopectineal orifice. It offers postoperative benefits in terms of reduced pain, faster recovery, earlier return to work and normal activity and better long-term comfort.

The laparoscopic inguinal hernia repairs, both TEP and trans-abdominal preperitoneal (TAPP), are looked upon as technically demanding procedures. Both have a steep ‘learning curve’ associated with their performance and complication rates have been shown to drop with increased surgical experience.[2–4] There is evidence to suggest that proper training and supervision can shorten the learning curve and reduce the complications and recurrences.[4–5]

The results of TEP repair have improved with time. The high volume centers have reported result outcomes which are comparable to, if not better than, the open hernia repair in terms of recurrence, chronic residual pain and quality of life.[5] This definitely suggests that surgeons who complete the learning curve can deliver results that are not only acceptable but also impressive and significantly better than open repair.[6–9] The improved outcomes can be attributed to assimilation of results of published literature and identifying factors that contribute to complications and failure of the repair. It is important to critically analyze each of these factors and help postulate surgical techniques that would reap good surgical outcomes.

The complication rate for laparoscopic repair of inguinal hernia ranges from less than 3%[6] to as high as 20%.[10–11] Complications that occur during a TEP repair include general complications that occur with any surgical procedure and anesthesia, mesh-related complications and those specific to the TEP procedure. Noteworthy intraoperative complications specific to TEP repair of inguinal hernias are:

## Vascular injury

- This can involve the iliac vessels, inferior epigastric vessels, spermatic vessels, muscular branches, vessels over the pubic arch (including corona mortis vein) or other vessels in the region.

## Visceral injury

- *Bowel injuries* can occur with trocar entry or during the course of dissection in large irreducible hernias, sliding hernias or with the use of electrodiathermy. The incidence of bowel injuries is greatly reduced, but sadly not completely eliminated, with TEP as compared to TAPP repair.[10]
- *Urinary tract injuries* are reported in laparoscopic inguinal hernia repair. These include bladder injuries and rarely even urethral rupture.

## Nerve injuries

The myopectineal orifice of Fruchaud has several nerves coursing it, viz, ilioinguinal nerve, iliohypogastric nerve, genito-femoral nerve with its medial genital (external spermatic nerve) and lateral femoral (lumbo-inguinal nerve) branches, femoral nerve and lateral femoral cutaneous nerve. All these are prone to injury, either during lateral dissection or during mesh fixation. It can result in long-term pain and discomfort.[12]

## Injury to cord structures

The vas deferens can be damaged or transected, as can the testicular vessels, during the course of dissection.

Complications can occur at every step of the operation, even though some of them are occasionally reported and are without any significance. However, it is important to analyze all of them chronologically, so that we can define methods to prevent them or tackle them once they occur.

A risk reduction strategy is required to improve the clinical outcome and this must be adopted during the following surgical steps:

1. Placement of the first trocar
2. Placement of the working port
3. Dissection of the preperitoneal space
4. Dissection of the hernial sac
5. Mesh placement and fixation
6. Closure

## 1. Placement of the first trocar

In laparoscopic surgery and when discussing TAPP, almost all injuries that are expected from the first trocar entry can be attributed to a sharp instrument like a Veress needle or sharp trocar entering the peritoneal cavity and causing potentially fatal vascular or visceral injury.[10–11] This type of injury is not common in a TEP repair, in which the preperitoneal space is entered under direct vision and a preperitoneal domain is maintained during the procedure. Few authors[13] advocate the use of Veress needle in the suprapubic area to create a working space, followed by a blind insertion of the first trocar; and this maneuver can lead to an inadvertent injury of the bladder or bowel. It may also rarely be encountered when a previous laparotomy (especially with an infraumbilical midline incision) results in scarring with distortion of tissue planes and visceral adhesions to the anterior abdominal wall.

We strongly recommend an open entry method for TEP, wherein the skin is incised and subcutaneous fat dissected to bare the anterior rectus sheath. The fascia is then incised and rectus muscle fibers split to expose the posterior rectus sheath. This plane is then maintained and a space is created inferiorly towards the pubic symphysis, using either gauze or a finger or balloon specially designed for this purpose. A Hasson's trocar is then introduced in this plane followed by the optics to confirm the plane, which is subsequently insufflated with carbon dioxide gas at 8–10 mmHg.

During this step, the posterior rectus sheath can be inadvertently breached along with the peritoneum, which can result in pneumoperitoneum. This pneumoperitoneum can have a pressure effect on the anterior abdominal wall, thereby minimizing the operating space and making further dissection difficult. It is important to identify and repair these tears early to facilitate smooth surgery. In case of previous surgical scarring, extra caution and vigilance is required at every step.[14] In select cases, it may be safer to opt for elective open repair, if deemed necessary.

## 2. Placement of working trocars

Two working trocars, each of 5 mm, are all that is required for the TEP repair in addition to a 10-mm infraumbilical trocar for the camera. These two trocars are usually placed in the midline, at one-third and two-thirds the distance from the umbilicus to pubic symphysis respectively. In our experience,[5] this allows the repair of bilateral hernias without insertion of further trocars. A different technique is described by some authors, viz, positioning a lateral or paramedian trocar as this configuration is considered more ergonomic; however, this will imply a wider lateral dissection and in case of bilateral hernias the necessity to insert a contralateral trocar. All trocars must be introduced under direct vision, taking care not to thrust the sharp tip into the bladder, peritoneum or underlying bowel. The inferior epigastric vessels must be avoided, especially if the trocars are not inserted along the midline. These vessels can bleed significantly if damaged. Direct pressure tamponade or electrodiathermy is usually sufficient, though rarely a transfascial or intracorporeal suture may be necessary. Peritoneal tears are discussed *vide* infra. Any visceral injury at this stage may require immediate conversion to a laparotomy.

## 3. Dissection of the preperitoneal space

Dissection of the preperitoneal space is done either with special balloon dissectors, mentioned earlier or with blunt dissection using the telescope and the two working trocars. The space must be clearly defined, starting with the pubic arch and symphysis in the midline. The bladder should be gently dissected off the pubis and rectus muscle superolaterally. During the dissection of the bladder from the rectus muscle and pubic symphysis, difficulty may be encountered if the patient has undergone previous surgery involving the prevesical space of Retzius (e.g., previous TEP, prostate surgery, etc.). The bladder is particularly vulnerable when it makes up part of the direct sac. Adhesions in this plane can result in bladder injury. It is important that such procedures are carried out by experienced surgeons with gentle and meticulous dissection, minimizing the use of electrodiathermy. If an injury does occur, it is important to identify it on table and deal with it. The presence of urine in the dissection plane or a sudden decompression of a distended bladder should arouse suspicion. The bladder tear should be sutured in two layers with absorbable material, using additional ports if necessary. The temptation to endoloop such tears should be resisted. The bladder should be decompressed postoperatively with a urinary catheter. It is helpful to preoperatively catheterize patients undergoing bilateral repair or those in which a lengthy procedure is anticipated, on account of large hernial sacs, irreducibility, previous surgical scarring or the surgeon and/or operative assistants being in the early phase of the TEP learning curve. Complex bladder injuries, including urethral tears, will more often than not require laparotomy and urological repair.

The medial dissection is done in the zone between the inferior epigastric vessels on either side, allowing adequate contralateral dissection. The ipsilateral set of inferior epigastric vessels is reflected upwards (anteriorly) with the help of one 5-mm blunt dissector, while the other dissector opens up the plane laterally. Bleeding from these vessels has been discussed *vide* supra. The area bound by the vas deferens superomedially and gonadal vessels superolaterally constitutes the so-called ‘triangle of doom.’ It houses the common iliac vessels. All dissection must stay clear of this zone and be carried out superior to it. Injury to these vessels can be fatal and usually requires an urgent laparotomy and vascular repair.

The lateral dissection (Bogros' space) is done beyond the anterior superior iliac spine, all the way up to the psoas muscle inferolaterally, thereby exposing the nerves in the ‘lateral triangle of pain.’ The musculopectineal orifice of Fruchaud has several nerves coursing it. These include ilioinguinal nerve, iliohypogastric nerve, genitor-femoral nerve with its medial genital (external spermatic nerve) and lateral femoral (lumbo-inguinal nerve) branches, femoral nerve and lateral femoral cutaneous nerve. Injury to the nerves at this point can result in postoperative discomfort and chronic groin pain. If an injury occurs inadvertently, the nerve should be infiltrated with a local anesthetic. This however, does not ensure uneventful sequelae. The only effective management of these nerve injuries is their prevention.

## 4. Dissection of hernial sac

This is a vital step of the procedure. It is important to identify all potential hernial sacs in the myopectineal orifice. Failure to recognize a complex hernia intraoperatively accounts for approximately 15% of failed repairs.[2,15] The iliopubic tract must be completely bared. The thinned out transversalis fascia (commonly referred to the pseudosac) and the peritoneum should be delineated fully, identifying any indirect and direct components to the hernia. A rational blend of predominantly blunt and minimal sharp dissection must be carried out, with sparing use of electrodiathermy. An indirect sac must be carefully separated from the spermatic cord and its contents. If a lipoma is identified accompanying the cord, it must be meticulously dissected out to prevent any recurrence. A sliding hernia or a fully or partially irreducible hernial sac can predispose to visceral injury. Bowel injury merits laparotomy. The hernia repair may be deferred depending on the amount of contamination. Whenever there is spillage of bowel contents, we recommend laparotomy and repair of bowel only in the primary sitting. The mesh repair should be deferred for another day, after an appropriate interval and recovery.

If the indirect sac is long or complete, it is often wise to circumcise and ligate (using a pre-made absorbable loop) the sac in the inguinal canal. The distal end must be left open to avoid a hydrocele. Unnecessary dissection can result in lymphatic destruction with seroma or formation or even bleeding intraoperatively with subsequent hematoma formation in the postoperative period. The seroma or hematoma can occur in the groin or go down to the scrotum. Extensive cord dissection should be supplemented with a good scrotal compressive dressing in the early postoperative period.

Rough handling of the cord structures can also cause bleeding from the testicular and cremasteric vessels. This can also preclude to hematoma formation and potential orchitis or testicular atrophy. Firm pressure usually controls this bleed and diathermy is sparingly used. The vas deferens can be inadvertently transected during dissection of the cord structures. Unilateral injury may not have any consequences, but all attempts must be made to repair it with an end-to-end anastomosis, with a conversion to open surgery if required. In the elderly, it can be safely ligated or clipped *in situ.*

Any breach in the peritoneum, including the indirect sac or pseudosac, should be avoided at this stage. If a tear does occur, it results in escape of insufflated gas to the intraperitoneal cavity. This not only affects the respiratory dynamics but also results in loss of working domain, making further dissection difficult and possibly dangerous. Pneumoperitoneum can also precipitate postoperative ileus. All such tears should be closed, usually with an absorbable endoloop. Larger tears may need multiple absorbable loops or intracorporeal sutures. At times, the pneumoperitoneum may warrant the placement of a Veress needle in the left subcostal position (Palmer's point) to deflate the gas and restore the domain. A missed tear can result in omental or intestinal herniation[16] through the defect, with potential intestinal obstruction, incarceration, strangulation and delayed fistulization.

## 5. Mesh placement and fixation

Ample dissection in the extraperitoneal space allows proper placement of an appropriate mesh in a TEP repair. The choice of mesh may vary, but commonly used is the flat polypropylene mesh. Variants of this mesh, with less content of polypropylene or with partially absorbable components, are also being used, with the rationale of light weight (less foreign body reaction), better handling and better long-term comfort. The flat mesh used should be at least 15 cm in width and 12–13 cm in height to cover the entire myopectineal orifice. The correct size of the mesh is important to prevent a late recurrence due to an eventual ‘shrinkage’ of the prosthesis.[17] This necessitates adequate dissection to allow the mesh to fit in, without folding or rolling at the edges. Anatomical meshes are of different shapes, designs and sizes. All of them also require this dissection to accommodate them suitably.

Irrespective of the prosthesis used, all meshes should be opened and handled with utmost aseptic care. They should be delivered in the space through an appropriately sized trocar (usually 10–12 mm), ideally through a reducer sleeve, to avoid any fraying or damage to the mesh. Slitting of the mesh is discouraged, as it is a potential space for recurrences, as reported in the early series of the laparoscopic repair.

While selecting the mesh type and size, it is important to define the hernial defect. The mesh should cover all potential hernial sites apart from the defined defect. This includes the direct hernia in the Hasselbach's triangle, indirect hernia lateral to the inferior epigastric vessels and along the inguinal canal, femoral hernia in the femoral canal inferior to the Cooper's ligament and obturator hernia in the obturator canal.

A flat mesh in the preperitoneal space is in constant danger of being displaced by intra-abdominal forces, before the fibrosis and scarring allows it to be incorporated as part of the posterior wall of the inguinal canal. During this period, inadequate mesh fixation can result in recurrence. The medial edge of the mesh is particularly prone to being displaced more than the lateral edge. When this displacement is enough to expose the medial part of the inguinal canal, including the Hasselbach's triangle, a recurrence is inevitable. This highlights the need to maintain the mesh in position. Perhaps, the commonest method advocated today is to use an anchorage device like tacks or staples to fix the mesh. It is important to have a good positioning of these anchorage devices, viz, Cooper's ligament, superior and medial to the direct defect, etc. It is strongly recommended to avoid any tacks or staples below the iliopubic tract, especially avoiding the triangles of doom and pain. No lateral fixation is advocated, to avoid inadvertent damage to the nerves. If a nerve is accidentally injured and this is identified on table, the anchorage device should be removed and a local anesthetic infiltrated in the region. Misplaced staple devices can also account for nerve irritation and injury. Postoperative pain and paresthesias can be a menace and haunt both the patient and the surgeon, with no cure guaranteed even with the utmost of corrective measures.

Several studies have recommended no fixation but have been found wanting.[18–19] Tissue glues are being used to fix the mesh in place, with encouraging early results.[20] Suturing of the mesh has also been described, but it requires expertise. Alternatively, anatomical meshes have also been designed for this purpose, which do not require any additional fixation. They conform to the space by virtue of their shape and prevent the mesh from migrating.[21]

## 6. Closure

After the mesh is appropriately placed and fixed, the operating field should be examined to rule out mesh displacement or folding. All hernial sacs and peritoneal folds must be defined. The mesh must be tucked inferior to the pseudosac and peritoneum and the indirect sac must be posterior to the mesh. Peritoneal tears must be looked for and dealt with appropriately, as mentioned earlier. Working trocars should be removed under vision, to rule out bleeding from the rectus muscle or vessels in the abdominal wall. Hemostasis much be achieved. The anterior rectus sheath (fascia) should be closed under direct vision. Scrotal or groin compression should be given, if necessary.

Good surgical technique, at every step of the procedure, can be mastered with time. Once the learning curve is through, satisfactory outcomes can be delivered with results comparable and even better than conventional repair. It is important for surgeons in the learning curve to be cautious and ideally supervised by experts so that potentially fatal complications do not put the procedure in disrepute.
